# Translational Windows in Chordoma: A Target Appraisal

**DOI:** 10.3389/fneur.2020.00657

**Published:** 2020-07-08

**Authors:** Samantha E. Hoffman, Sally A. Al Abdulmohsen, Saksham Gupta, Blake M. Hauser, David M. Meredith, Ian F. Dunn, Wenya Linda Bi

**Affiliations:** ^1^Center for Skull Base and Pituitary Surgery, Harvard Medical School, Brigham and Women's Hospital, Boston, MA, United States; ^2^Department of Pathology, Harvard Medical School, Brigham and Women's Hospital, Boston, MA, United States; ^3^Department of Neurosurgery, University of Oklahoma College of Medicine, Oklahoma City, OK, United States

**Keywords:** chordoma, genomics, immunology, targeted therapy, checkpoint inhibition

## Abstract

Chordomas are rare tumors that are notoriously refractory to chemotherapy and radiotherapy when radical surgical resection is not achieved or upon recurrence after maximally aggressive treatment. The study of chordomas has been complicated by small patient cohorts and few available model systems due to the rarity of these tumors. Emerging next-generation sequencing technologies have broadened understanding of this disease by implicating novel pathways for possible targeted therapy. Mutations in cell-cycle regulation and chromatin remodeling genes have been identified in chordomas, but their significance remains unknown. Investigation of the immune microenvironment of these tumors suggests that checkpoint protein expression may influence prognosis, and adjuvant immunotherapy may improve patient outcome. Finally, growing evidence supports aberrant growth factor signaling as potential pathogenic mechanisms in chordoma. In this review, we characterize the impact on treatment opportunities offered by the genomic and immunologic landscape of this tumor.

## Introduction

Chordomas are locally aggressive tumors arising from fetal notochord remnants with an average annual incidence of 0.088 per 100,000 in the United States (1). These tumors frequently occur at the skull base, followed by sacrum and vertebral bodies, with increasing incidence with age (1). Gross total resection followed by adjuvant radiation offers long-term disease control in many patients (2, 3). However, encasement of vital neurovascular structures and concealed nests of tumor may hinder complete eradication, leading to repeated recurrences following subtotal resection (4, 5). They are also prone to seeding and late metastasis (6). Once recurrent, chordomas are extremely refractory to cure. Chemotherapy has been used for recurrent and metastatic disease, without any consistent efficacy thus far (7, 8).

The difficulties of managing aggressive and refractory chordoma have motivated study of the biological underpinnings of this disease. Genome-scale sequencing approaches and a resurgent interest in tumor immunology have been pivotal in expanding our understanding of chordoma. In this review, we discuss recent advances in the understanding of chordoma biology with a focus on its immune and extracellular microenvironment.

## Histopathologic Characteristics

**Figure 1:**
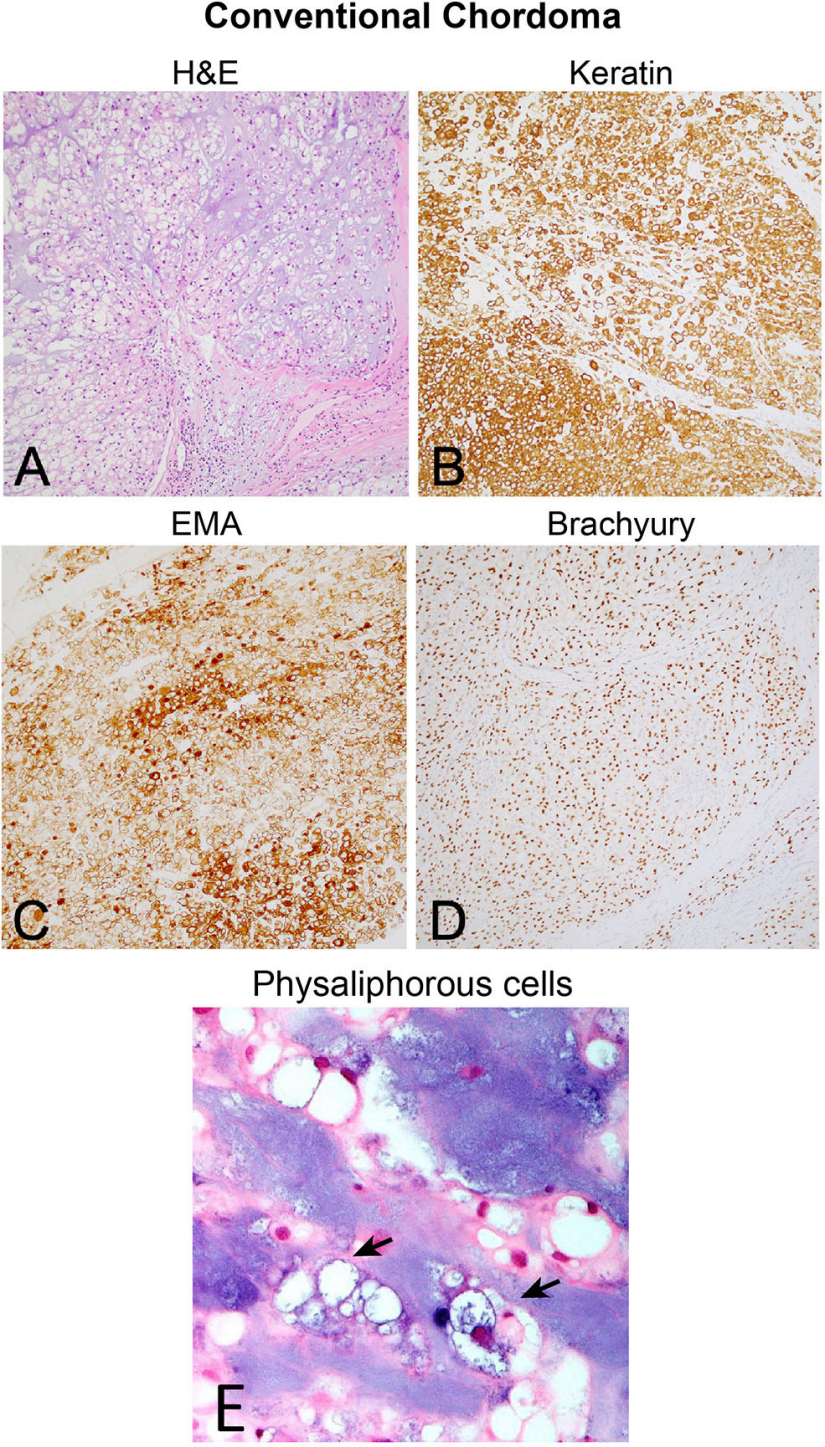
**(A)** Conventional chordoma showing classic histologic features of large, epithelioid tumor cells with eosinophilic cytoplasm containing multiple vacuoles arranged in nests and cords in an overall lobular growth pattern. Tumor cells are reliably positive for **(B)** keratins and **(C)** EMA. S100 staining may be seen in select cases but is not ubiquitous (not shown). **(D)** Nuclear positivity for brachyury is highly specific and observed in the vast majority of cases. **(E)** Physaliphorous cells (arrows) display abundant, “bubbly” cytoplasm with small, round nuclei on higher magnification. Large and small cells can be seen in the background of blue, myxoid stroma.

**Figure 2:**
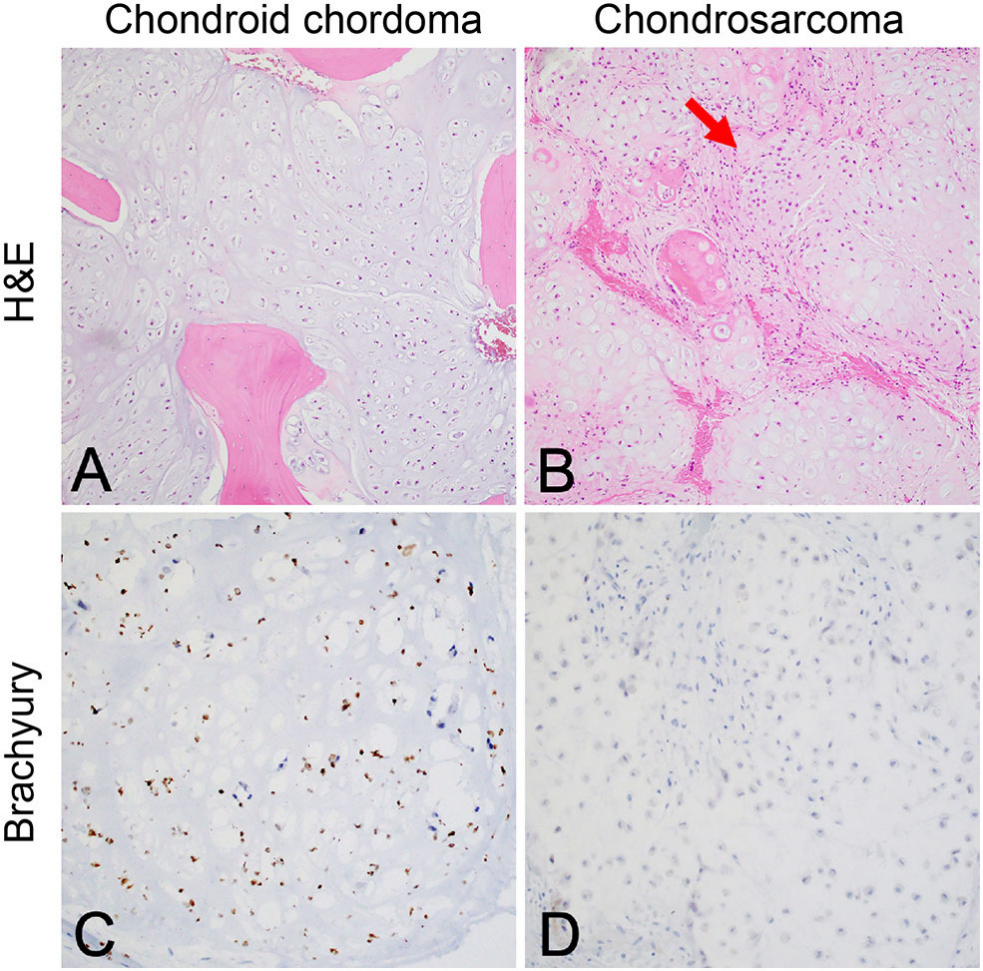
**(A)** Chondroid chordoma characteristically contains regions forming hyaline cartilage with tumor cells distributed individually in lacunar spaces that greatly resemble **(B)** low-grade chondrosarcoma, which classically feature hypercellular hyaline cartilage lobules with a permeative growth pattern. Chondrosarcomas of the skull base in particular have variably prominent myxoid areas (arrow) that resemble chondroid chordoma. **(C)** Brachyury nuclear positivity confirms the diagnosis of chordoma, while being **(D)** consistently negative in chondrosarcoma.

**Figure 3:**
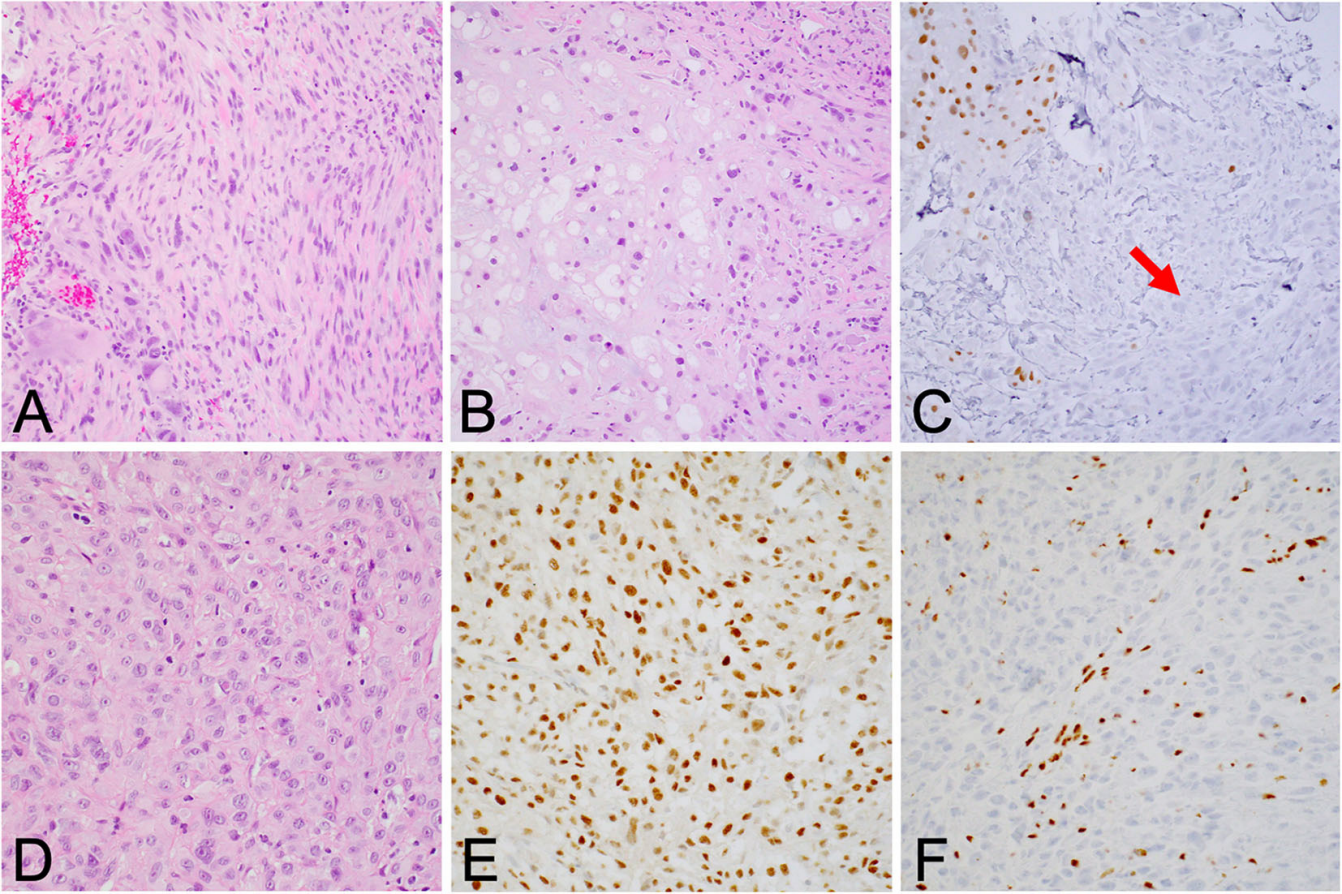
**(A)** Dedifferentiated chordoma contains regions of high-grade sarcoma, frequently juxtaposed with regions of conventional chordoma **(B)**. **(C)** Brachyury positivity is often negative in the dedifferentiated component (arrow). **(D)** Poorly differentiated chordoma exhibits higher cellularity than conventional chordoma and occasionally shows rhabdoid morphology. **(E)** Brachyury is reliably positive, and **(F)** SMARCB1 (INI-1) loss in tumor cells is characteristic of this entity.

Although the notochord is derived from the ectoderm, chordoma shows both epithelial and mesenchymal differentiation. Their spectrum of inter- and intratumoral histological patterns render their level of differentiation and clinical behavior challenging to predict (9). The World Health Organization classification of chordoma subtypes includes conventional chordoma (Figure 1), chondroid chordoma (Figure 2), and dedifferentiated chordomas (Figure 3). Conventional chordomas show three neoplastic cell types in a myxoid stroma: (1) intermediate-sized physaliphorous cells are the main proliferating cell type and appear as distinctive vacuolated cells in cords and lobules, (2) large cells with prominent vesicular nuclei, and (3) small cells with pyknotic nuclei. Dedifferentiated chordomas are associated with poorer prognosis compared to conventional and chondroid subtypes, while chondroid chordoma is associated with worse survival than classic chordoma (10). Importantly, chondroid chordoma should be distinguished from chondrosarcoma based on brachyury immunopositivity, as chondrosarcoma has a significantly more indolent growth rate than any chordoma, and therefore may merit different treatment considerations than chordoma itself (11).

## Genomic Alterations

Some *de novo* chordomas harbor chromosomal changes, which are associated with poorer prognoses (12, 13). The most common genomic alterations include large copy number losses in chromosomes 1p, 3, 4, 9, 10, 13, and 14; chromosomal deletions are more common than gains (14). Wide-scale deletions in 1p and 9p specifically have been associated with worse prognosis even after resection and radiation (15). Recurrence is not associated with increased copy number loss or gain compared to primary tumors (16).

### Brachyury

Brachyury is a protein whose expression is the diagnostic hallmark of chordoma, regardless of subtype (17). Encoded by the *TBXT* or *T* gene on chromosome 6q, brachyury binds palindromic *T* sites to exert DNA-regulatory effects. Germline duplications of *T* are associated with familial chordomas, while somatic tandem *T* duplications have been identified in sporadic chordoma (18). Chordomas with increased brachyury expression are associated with poorer prognosis (18).

A member of the T-box protein family, brachyury is a transcription factor that regulates notochord cell fate determination during development (17). Brachyury is also a mediator of the epithelial-to-mesenchymal transition by downregulating E-cadherin expression, explaining both the heterogeneous histology and the metastatic tendency of chordomas (17).

The ubiquitous brachyury expression in chordoma makes it an attractive therapeutic target; however, its nuclear localization has barred access to targeted inhibitors, setting the stage for immunotherapeutic approaches (19). Targeting brachyury with a recombinant yeast vaccine has been found to activate human T cells *in vitro* and enhance immune response in phase I trials; brachyury vaccination is now being investigated in a phase II trial as well (Table 1) (20).

**Table 1:** Active trials for surgical, radiation, and medical therapies for chordoma (extrapolated from ClinicalTrials.gov; December 30, 2019).

**Official study title**	**Treatment**	**Phase**	**# Participants**	**Sponsor**	**Projected completion date**	**Trial registration no**.
**IMMUNOTHERAPY**						
Nivolumab with or without stereotactic radiosurgery in treating patients with recurrent, advanced, or metastatic chordoma	Nivolumab, stereotactic radiosurgery	I	33	Sidney Kimmell Comprehensive Cancer Center at Johns Hopkins	March 2021	NCT02989636
Nivolumab and Ipilimumab in treating patients with rare tumors	Nivolumab, Ipilimumab	II	707	National Cancer Institute	August 2021	NCT02834013
Nivolumab and Relatlimab in treating participants with advanced chordoma	Nivolumab, Relatlimab	II	20	UCLA/Jonsson Comprehensive Cancer Center	April 2022	NCT03623854
Talimogene Laherparepvec, Nivolumab, and Trabectedin for sarcoma (TNT)	Talimogene Laherparepvec, Nivolumab, Trabectedin	II	40	Sarcoma Oncology Research Center, LLC	December 2022	NCT03886311
**SMALL MOLECULE INHIBITORS**						
Nilotinib with radiation for high risk chordoma	Nilotinib	I	29	Massachusetts General Hospital	December 2019	NCT01407198
Afatinib in locally advanced and metastatic chordoma	Afatinib	II	40	Leiden University Medical Center, Netherlands	December 2019	NCT03083678
CDK4/6 inhibition in locally advanced/metastatic chordoma	Palbociclib	II	43	University Hospital Heidelberg, Germany	July 2021	NCT03110744
Anlotinib Hydrochloride vs. Imatinib Mesylate in locally advanced, unresectable or metastatic chordoma (CSSG-03)	Anlotinib Hydrochloride	II	60	Peking University People's Hospital, China	December 2021	NCT04042597
A phase II, multicenter study of the EZH2 inhibitor Tazemetostat in adult subjects with INI1-negative tumors or relapsed/refractory synovial sarcoma	Tazemetostat	II	250	Epizyme, Inc.	February 2022	NCT02601950
**NATURAL HISTORY/OBSERVATIONAL**						
Children and adults with chordoma	Natural history	N/A	300	National Cancer Institute	December 2029	NCT03910465
Genetic clues to chordoma etiology: a protocol to identify sporadic chordoma patients for studies of cancer-susceptibility genes	Observational	N/A	400	National Cancer Institute	N/A	NCT01200680
**SURGERY, RADIATION, OR CHEMOTHERAPY**						
Pemetrexed for the treatment of chordoma	Pemetrexed	I	15	John Wayne Cancer Institute	May 2023	NCT03955042
BN brachyury and radiation in chordoma	BN brachyury, radiation	II	29	Bavarian Nordic	January 2021	NCT03595228
Improvement of local control in skull base, spine and sacral chordomas treated by surgery and proton therapy targeting hypoxic cells revealed by [18F]FAZA) PET/CT tracers (PROTONCHORDE01)	18F FAZA, surgery, proton therapy	II	64	Institut Curie, France	September 2022	NCT02802969
Ion Irradiation of Sacrococcygeal Chordoma (ISAC)	Proton radiation, carbon ion radiation	II	100	Heidelberg University	June 2023	NCT01811394
Trial of Proton vs. Carbon Ion Radiation therapy in patients with chordoma of the skull base	Carbon ion radiation, proton radiation	III	319	Heidelberg University	August 2023	NCT01182779
Proton radiation for chordomas and chondrosarcomas	Proton therapy	N/A	50	Abramson Cancer Center of the University of Pennsylvania	December 2019	NCT01449149
A study of IMRT in primary bone and soft tissue sarcoma (IMRiS)	Intensity modulated radiotherapy	N/A	200	University College London, United Kingdom	March 2021	NCT02520128
Sacral Chordoma: Surgery vs. Definitive Radiation therapy in primary localized disease (SACRO)	Surgery, radiation therapy	N/A	100	Italian Sarcoma Group	September 2022	NCT02986516
Randomized Carbon Ions vs. Standard Radiotherapy for radioresistant tumors (ETOILE)	Carbon ion radiation, X-ray radiotherapy, proton radiation	N/A	250	Hospices Civils de Lyon, France	May 2024	NCT02838602

### Cell Cycle Regulation (CDKN2A, PTEN)

Upregulation of other T-box genes in malignancy is thought to repress cell-cycle regulators like Cyclin-dependent kinase inhibitor 2A (*CDKN2A*). The *CDKN2A* gene encodes two tumor suppressor proteins that inhibit progression through the cell cycle; mutations in this gene therefore result in an uncontrolled rate of cell proliferation and differentiation via activation of cyclin-dependent kinases 4 and 6 (CDK4/6). Similarly, the phosphate and tensin homolog (*PTEN*) gene also encodes for a tumor suppressor protein and regulates cell growth (**Figure 5**).

Genomic analysis of chordoma samples reveals widespread cell cycle dysregulation (14, 21, 22). Both homozygous and heterozygous copy number losses of *CDKN2A* have been identified in chordomas (14, 18, 21, 22). Of note, tumors with copy number losses in *CDKN2A* and *PTEN* also harbored point mutations in those genes, though no activating mutations were identified (21).

The success of CDK4/6 inhibitors in other malignancies has motivated their application to chordomas. Treatment of a CDKN2A-deleted chordoma cell line *in vitro* with palbociclib, for example, resulted in significant growth inhibition (23). Preserved CDK4/6 expression may serve as a potential biomarker for future screening.

Many chordomas feature *PTEN* loss, which may drive oncogenesis both through loss of tumor suppression and an increase in PD-L1 expression (7). To date, no studies have examined existing molecular therapies for chordomas with *PTEN* loss. Instead, *PTEN* upregulation has been investigated as a sensitizing factor to improve response to *PDGFR* and *mTOR* pathway inhibitors in these tumors (24, 25).

### Chromatin Remodeling

The pathogenesis of chordoma may involve DNA-level dysregulation that promotes oncogene expression or tumor suppressor silencing. A cardinal regulator of chromatin organization and subsequent regulation of gene expression is the Switch/Sucrose Non-Fermentable (SWI/SNF) protein complex. Mutations of SWI/SNF genes result in a loss of chromatin regulation, facilitating neoplastic development via uncontrolled DNA replication and subsequent cell proliferation.

The *SMARCB1* gene is a SWI/SNF component that is thought to serve a tumor suppressor role by regulating the histone methylation activity of the transcription factor EZH2 (26). SMARCB1 loss is the defining marker of poorly differentiated chordomas (PDCs), a highly aggressive subtype distinct from dedifferentiated chordomas that occur most frequently in children and at the skull base. Specifically, PDCs display a loss of nuclear *SMARCB1* expression and homozygous deletions of *SMARCB1*, implicating SWI/SNF aberrations in their rapidly progressive course (21, 27–29).

In other chordoma subtypes where SMARCB1 was downregulated but not lost, upregulation of microRNAs associated with transforming growth factor β signaling (TGF- β) was observed (30). TGF-β signaling may drive chordoma progression and survival through both augmented bone formation (31) and anti-inflammatory effects (32).

Mutations in three other SWI/SNF members—*PBRM1, SETD2, ARID1A*—have been identified as potential drivers in chordomas (18, 29); however, the relevance of these mutations to chordoma etiology remains to be elucidated.

## Immune Microenvironment

The immune microenvironment offers a therapeutic window for chordomas since there are no reliable molecular markers to predict clinical outcome and drug response in these tumors (Table 2) (45). The tumor microenvironment is often more genetically stable than tumor cells, which can rapidly mutate and acquire drug resistance. A lack of successful treatment options and sparse genetic drivers has driven exploration of immunomodulation strategies such as vaccination with tumor-specific neoantigens, chimeric antigen receptor T-cell (CAR-T cell) engineering, and blocking immune checkpoints.

**Table 2:** Completed clinical trials for targeted therapies in chordoma.

**References**	**Regimen**	**Mechanism**	**Study design**	**# Pts**	**SD**	**MR**	**PR**	**CR**	**PD**	**Median PFS**	**PFS6**	**Median OS**
(33)	Imatinib	PDGFR TKI	Retrospective	48	34	0	0	0	12	9.9 months	65%	30 months
(34)	Imatinib, sorafenib, erlotinib, sunitinib, temsirolimus	PDGFR TKI, PDGFR/VEGFR TKI, EGFR TKI, PDGFR/VEGFR TKI, mTOR TKI	Retrospective	80	58	0	5	0	10	9.4 months	NA	4.4 years
(35)	Sunitinib	PDGFR/VEGFR TKI	Phase II	9	4	0	0	0	5	NA	NA	NA
(36)	Sorafenib	PDGFR/VEGFR TKI	Phase II	27	12	0	1	0	1	NA	85.30%	NA
(37)	Lapatinib	EGFR TKI	Phase II	18	15	0	0	0	3	8 months	NA	25 months
(38)	Imatinib	PDGFR TKI	Phase II	50	21	9	1	0	9	9.2 months	NA	34.9 months
(39)	Dasatinib	SRC TKI	Phase II	32	NA	NA	NA	NA	NA	6.3 months	54%	21.6 months
(40)	Imatinib, everolimus	PDGFR TKI, mTOR TKI	Phase II	43	37	0	1	0	4	14	N/A	47.1
(41)	Linsitinib, erlotinib	IGF-1R TKI, EGFR TKI	Phase I	95	33	0	5	0	37	NA	NA	NA
(42)	Imatinib	PDGFR TKI	Phase I	7	NA	NA	NA	0	NA	10.2 months	NA	NA
(43)	Imatinib, sirolimus	PDGFR TKI, mTOR inhibitor	Phase I	10	7	0	1	0	1	NA	NA	NA
(44)	Nilotinib	PDGFR TKI	Phase I	23	NA	NA	NA	NA	NA	58.15	N/A	61.5

*SD, Stable disease; MR, Minor response; PR, Partial response, CR, Complete response, PR, Progressive disease per RECIST analysis*.

Though chordomas display an elevated immune infiltration, the immune effectors are likely generating an anti-inflammatory tumor microenvironment (46). For instance, infiltrating macrophages in chordomas are predominantly M2 (anti-inflammatory) macrophages; elevated CD47 expression on chordoma cells also downregulates pro-inflammatory macrophage activity (47).

Cytokine signaling generated by the immune microenvironment may augment the malignant behavior of these tumors. Tumor necrosis factor alpha (TNF-α) is an inflammatory cytokine produced by tumor-associated macrophages that can promote tumor growth and progression. Leukemia inhibitory factor (LIF) is a cytokine in the interleukin-6 family whose functions include regulating proliferation of cancer stem cells, like those often found in chordoma (48). Expression of either TNF-α or LIF in chordoma cell lines promoted cell migration, invasive capabilities, and anchorage-independent growth, demonstrating their pro-tumoral and metastatic potential (49). Exposure of chordomas to TNF-α upregulates not only its own gene expression but also *LIF* expression, and vice versa; both cytokines are correlated with increased tumor size, implicating these factors in chordoma growth (49, 50).

Immune cell signaling also induces alterations in gene expression in chordoma cells. Epithelial marker expression was downregulated while mesenchymal marker expression was upregulated following exposure to TNF-α, implicating TNF-α in the chordoma epithelial-mesenchymal transition (49). Differential gene expression analysis post-TNF-α exposure demonstrated upregulation of the phosphoinositide 3-kinase (PI3K)/Akt, Ras, and Ras-related protein 1 (Rap1) signaling pathways, implicated in progression of chordomas and other malignancies, respectively (49, 51). Pro-angiogenic and anti-apoptotic pathways were also upregulated with longer TNF-α exposure, which may contribute to the survival and metastatic potential of chordomas (49).

The chordoma microenvironment can impact treatment efficacy by limiting the penetration of pro-inflammatory effectors and altering the expression of putative therapeutic targets. LIF has been shown to inhibit CD8+ T-cell entry while increasing tumor-associated macrophage and regulatory T-cell entry into other tumors, decreasing the efficacy of immune checkpoint blockade (48). The abundance of extracellular matrix associated with chordomas may similarly contribute to lymphocyte distribution. Elevated stroma-tumor ratio was associated with decreased infiltration of effector T-cells and an elevated density of regulatory T cells within the tumor (52). TNF-α-exposed chordoma cells displayed increased programmed death-ligand 1 (PD-L1) but downregulated *T* expression; brachyury-targeted treatments may be impacted as a result (49, 50). Finally, resistance to cytotoxic chemotherapies also increases in these chordoma cells exposed to LIR and TNF-α (49, 50).

### Immune Checkpoint

Immune activation is physiologically downregulated by “immune checkpoints” pathways. Regulatory T cells express immune checkpoint ligands that bind to receptors on activated cytotoxic T-cells, inducing T-cell exhaustion. Immune checkpoint proteins normally inhibit autoimmune responses, but cancer cells can also upregulate these ligands to decrease inflammatory response to the tumor. Pharmacologic blockade of these immune checkpoints, known as checkpoint inhibitor drugs, have proven efficacious immunotherapies in several types of cancer, motivating their trial in chordoma (53).

### PD-1/PD-L1

**Figure 4:**
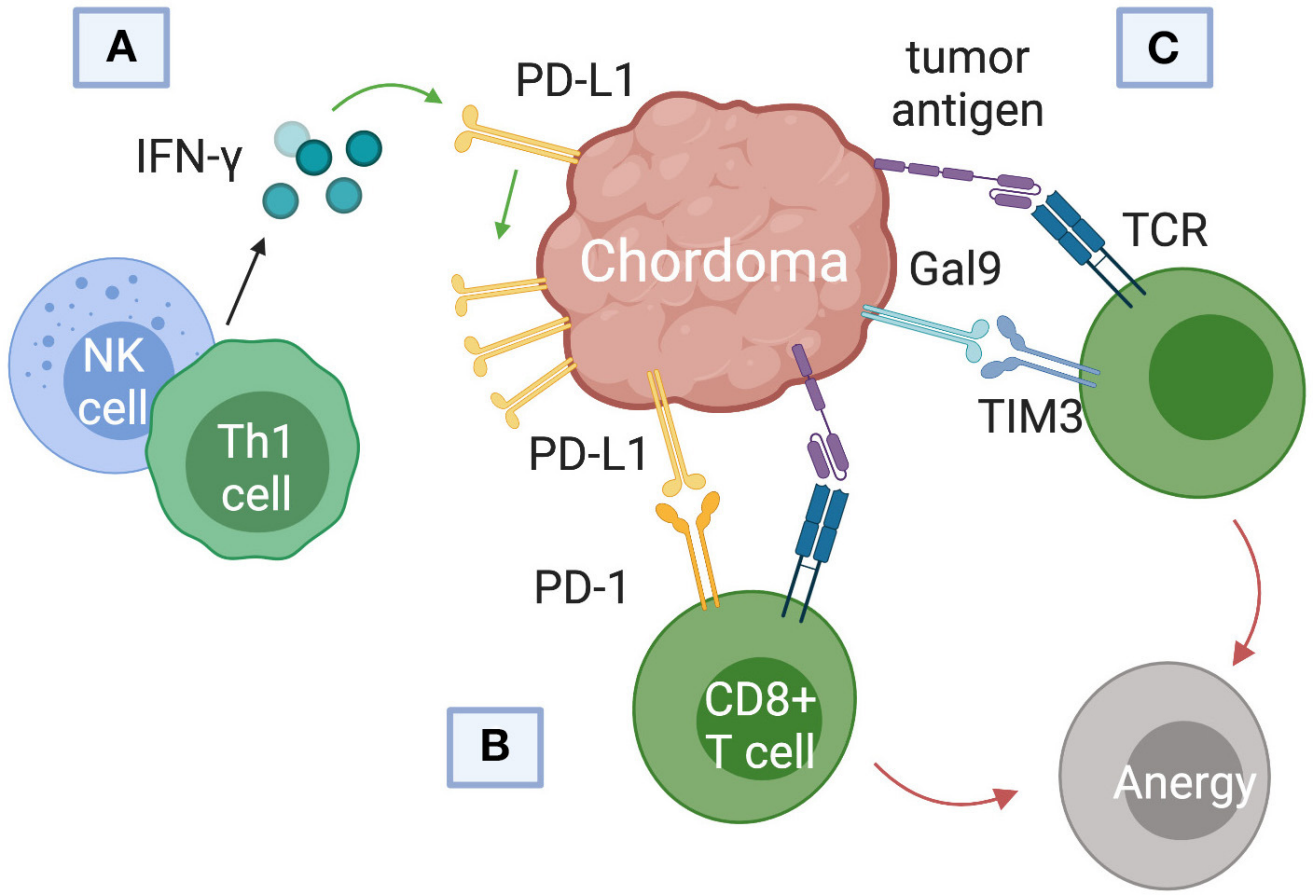
Immune checkpoint pathways implicated in chordoma pathogenesis and survival. **(A)** Interferon-gamma released from natural killer (NK) cells and helper CD4+ T (Th1) cells was found to upregulate programmed death ligand (PD-L1) expression in chordomas. **(B)** PD-L1 binds the programmed cell death protein 1 (PD-1) on cytotoxic T cells, inducing T cell anergy. **(C)** Chordomas also induce immunosuppression via the Gal9 immune-checkpoint protein binding to TIM3 on cytotoxic T cells.

Current immunotherapy trials of chordoma have focused on blockade of PD-L1, the immune checkpoint regulator responsible for suppressing regulatory T cell apoptosis (Figure 4). Whether or not chordomas harbor PD-L1 natively remains in contention, but chordoma cell lines uniformly upregulated PD-L1 expression after interferon-γ treatment (46). PD-L1 expression was also found on tumor-infiltrating macrophages and lymphocytes at the tumor-stroma interface (7). Chordomas with negative PD-L1 expression tended to have more PD-L1 positive tumor-infiltrating lymphocytes (TIL), and the prevalence of these cells correlated with metastatic potential (7).

The putative correlation between PD-L1 expression and malignancy augmented interest in checkpoint blockade in chordomas. Avelumab, an anti-PD-L1 monoclonal antibody, enhanced the immune-mediated killing of chordoma cells *in vitro*; upregulation of PD-L1 on the chordoma cells further increased their sensitivity to lysis (54). Cancer stem cells, which are resistant to radiation and chemotherapy, were also eliminated at higher rates by avelumab, suggesting that chordoma progression and recurrence may rely on immunologic escape mechanisms (54).

PD-L1 expression in chordomas may be further regulated by microRNAs, small non-coding RNA molecules responsible for post-transcriptional regulation of gene expression. Comparison of chordoma tissue with fetal notochords reveals significant microRNA dysregulation that may precede and augment the tumorigenic effects of PD-L1 upregulation (55). MiR-574-3p, for example, is a microRNA whose expression is negatively correlated with PD-L1 expression (56). Chordomas with low miR-574-3p and high PD-L1 expression were associated with higher muscle invasion, more tumor necrosis, and poorer patient outcomes (55). Elucidating the mechanism underlying this anticorrelation could augment checkpoint blockade implementation in these tumors.

### TIM3/Gal9

T-cell immunoglobulin and mucin-domain 3 (TIM3) has only recently emerged as a target of interest in chordoma immunotherapy and cancer therapy in general (57–59). First identified on cells of the adaptive immune system, TIM3 is also constitutively expressed on innate immune cells (59–61). TIM3 promotes tumor survival by binding to galectin-9 (Gal9) on tumor cells and inducing T cell exhaustion (Figure 4C). (61). Combining anti-TIM3 and anti-PD-1 regimens may better preclude immunotherapy failure compared to monotherapy, invigorating research into the TIM3/Gal9 pathway as a novel pharmaceutical target (62).

Only one study has investigated TIM3/Gal9 in the setting of chordoma, but its results are consistent with corresponding studies in solid and hematological malignancies (63). In a retrospective analysis of 93 skull base chordomas, the only parameter that independently predicted local recurrence-free survival on both univariate and multivariate analysis was the density of TIM3+ lymphocytes (63). Patients with low TIM3+ TILs in their tumors also had better overall survival compared to patients with high TIM3+ TIL density (63). A putative regulator of the Gal9 native to the chordoma microenvironment has been identified: microRNA 455-5p (miR-455-5p) (63). Tumors that are Gal9+/miR-455-5p-*high* are associated with longer overall survival than Gal9+/miR-455-5p-*low* tumors, suggesting an intrinsic downregulation mechanism that could be exploited for future immunotherapy design (63).

## Growth Factor Signaling

Growth factor signaling pathways are key modulators of cell growth, proliferation, and survival in normal biology. Aberrant and amplified cascades also play a significant role in tumor pathogenesis. Targeted inhibition of known oncogenic growth factors has driven chordoma clinical trials, and genomic analysis of these tumors has provided several new candidate pathways whose roles remain fully unexplored.

### PDGF and EGF Pathways

**Figure 5:**
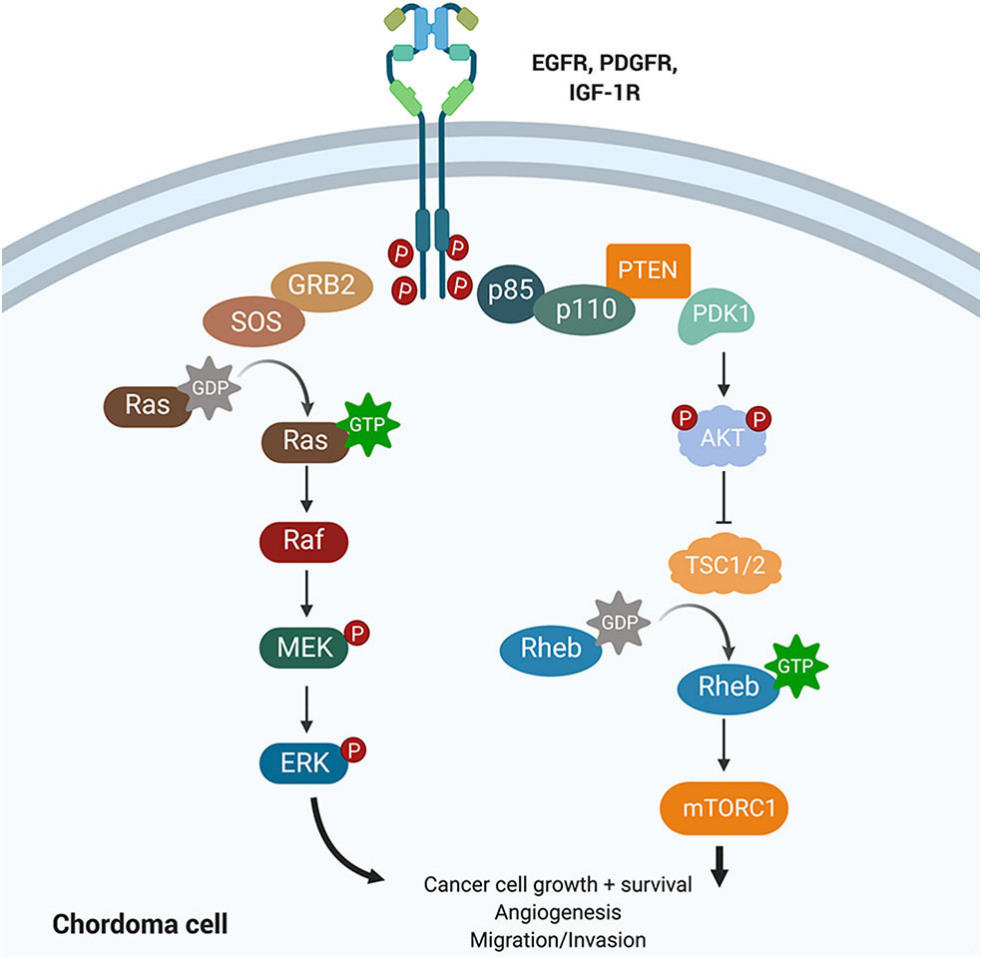
Growth factor signaling pathways implicated in chordoma pathogenesis. The PI3K/AKT and the RAS pathways lie downstream of EGFR, PDGFR, and IGF-1R and have been implicated in cancer promotion and progression. Upregulation of PI3K/AKT was associated with high brachyury expression in chordomas and may also regulate tuberous sclerosis comorbidity with chordomas. IGF-1R is a novel target whose activation was associated with poorer prognosis and increased tumor volume in chordomas.

Amplification of platelet-derived growth factor (PDGF) and epidermal growth factor (EGF) signaling has been shown to increase tumor hyperplasia and survival (Figure 5). Both PDGF and EGF bind to a receptor tyrosine kinase (PDGFR and EGFR), resulting in activation of downstream signaling cascades that regulate proliferation and differentiation. Tyrosine kinase inhibitors (TKIs) can selectively block PDGFR and EGFR phosphorylation and have revolutionized cancer management.

There is significant burgeoning interest in applying TKIs to chordomas (Tables 1, 2). Numerous clinical trials to date have investigated the use of PDGFR and EGFR TKIs as either monotherapies or in combination with other small molecule inhibitors (Table 1). Complete response was not achieved by RECIST analysis in any of the trials or retrospective analyses, with the majority of patients experiencing either stable or progressive disease.

### PI3K/Akt/mTOR Pathway

The phosphoinositide 3-kinase (PI3K)/Akt signaling pathway regulates cell cycle progression, cellular proliferation, survival, and cell growth (Figure 5). Activating mutations of this pathway, downstream of PDGF and EGF signaling, are some of the most commonly identified driver mutations in malignancy. For example, mutations in *mTOR* and the tumor suppressors *TSC1* and *TSC2*, components of the PI3K/Akt cascade, have been associated with the development of tuberous sclerosis complex syndrome (TSC) in children. The dual diagnosis of TSC and chordoma in several children motivated the study of the PI3K/Akt pathway in chordomas (64). TSC-associated chordomas emerge significantly earlier than other pediatric chordomas, and sacral chordomas are associated with a significantly better prognosis when concurrent with TSC (64).

Protein analysis reveals a possible mechanism underlying the TSC-chordoma association. Positive expression of phosphorylated AKT and TSC2 identified across chordoma samples indicates loss of tumor suppression stemming from activation of the PI3K pathway (65). Phosphorylated p70S6kinase expression (p-p70S6K), which lies downstream of TSC2 activation, was also identified; together, these data support the use of mTOR inhibitors to treat chordoma (65).

Analysis of chordoma cell lines not associated with TSC also revealed a correlation between PI3K/AKT pathway upregulation and brachyury expression (66). Cells from skull-base chordomas with high brachyury expression had significant upregulation of PI3K/AKT pathway genes compared to low-brachyury tumors (66). Treatment with PI3K/AKT pathway inhibitors resulted in decreased brachyury expression that corresponded with impaired cell growth (66). These results confirmed that PI3K/AKT pathway activation augmented brachyury expression (66). Importantly, *mTOR, TSC2, EGFR*, and *PDGFR* were not among the upregulated genes identified in the high-brachyury tumors (66).

Few clinical trials investigating PI3K/AKT/mTOR inhibition in chordomas exist to date; current trials implement mTOR inhibitors secondary to other small-molecule inhibitors (43). A small number of published case reports found neither rapamycin or everolimus monotherapy to be efficacious (34, 67), despite prior suggestion of rapamycin-responsiveness in patient-derived cell lines (68). A randomized investigation of these therapies may further clarify their efficacy in chordoma treatment.

### IGF-1 Pathway

Chordoma's osseous localization has prompted investigation of bone growth regulation as a possible pathogenic pathway. Interest in insulin-like growth factor 1 (IGF-1), one such growth mediator, in chordomas has been spurred by two features of the IGF family. First, IGF-1 effects strong mitogenic activity in bone, which, if dysregulated, could accelerate chordoma development. The IGF-1/IGF-1R axis is also easily targetable by existing small molecular inhibitors.

Emerging literature supports a relationship between IGF-1/IGF-1R expression and prognosis in chordomas. The majority of chordoma samples studied were positive for both IGF-1 and IGF-1R; the level of IGF-1R staining correlated with tumor volume (69). Phosphorylated-IGF-1R expression was also associated with decreased median progression-free survival (70). Activation of IGF-1R signaling may therefore contribute to chordoma growth or progression, presenting a potential new biomarker and targetable pathway (69, 70). Given that the AKT/mTOR pathway lies downstream of IGF-1/IGF-1R signaling (71) and the success of combined mTOR/IGF-1R inhibition in sarcomas (72), future clinical trials could benefit from looking at a similar combined approach to chordoma management. Clinical trials of IGF-1R inhibitors in chordoma have thus far comprised phase I studies of combined anti-IGF-1R/EGFR inhibition and suggest some efficacy to this approach (41, 73).

## Extracellular Matrix

Chordomas produce abundant extracellular matrix. High molecular weight melanoma-associated antigen expression is detected in 62% of chordomas, making them an attractive matrix target (74). Cathepsin K is a cysteine protease that is thought to play a role in osteoclast-mediated bone resorption. Its high expression in the invasion fronts of chordoma, compared to chorda dorsalis and chondrosarcoma controls, incriminates Cathepsin K in chordoma's infiltrative growth (75). Morphogens, signaling molecules that govern embryological tissue development in the process of morphogenesis, along with extracellular signals that regulate embryonic notochord development may also play key roles in establishing a microenvironment that promotes chordoma pathogenesis (76).

## Future Directions

Novel therapies for chordoma will need to demonstrate efficacy via clinical trials, but several disease and patient-intrinsic factors complicate the execution of these trials (77, 78). First, durability of long-term disease control with complete surgical removal of tumor and invaded bone followed by radiation remains salient for newly diagnosed chordomas. Clinical trials, therefore, center on recurrent and therapy-resistant chordomas for which effective control proves more challenging. The low incidence of these tumors, especially in the recurrent setting, leads to an even smaller population of eligible patients for accrual into diverse clinical trials, which may underpower the testing of any specific hypothesis. The increased average age of chordoma patients and associated comorbidities may further winnow this population following trial exclusion criteria. Lack of access to clinical trials and physician referral patterns may also impact patient enrollment in clinical trials (78). In general, chordoma remains understudied compared to more prevalent malignancies; efforts by patient advocacy groups like the Chordoma Foundation and research consortiums continue to raise awareness and funding for this rare disease.

Future clinical trials for chordoma will benefit from the adoption of innovative and integrative methods to overcome these obstacles (77, 78). Existing trials, such as the Bayesian design of the glioblastoma AGILE trial, may provide a model for efficient assessment of a large armament of therapy options in a rare disease cohort; optimal assignment of patients to clinical trials requires stratification based on clinical indication and prognostic biomarkers, which have yet to be fully elucidated in chordoma (79, 80).

Finally, increased understanding of the biological crosstalk within chordomas highlight a possible role for combination immunogenomic therapy moving forward. The tumor-intrinsic and microenvironmental heterogeneity of chordomas portends risk of emerging resistance to any rationally designed, molecularly targeted therapies, while therapies themselves have been shown to induce genomic alterations in multiple cancers. The promise of immunotherapies, either independently or synergistic with molecularly targeted therapies, presents a particularly promising adaptive strategy for future investigation.

In all, clinical and scientific investigations into chordoma hold promise for the identification of genetic, molecular, and immunomodulatory agents involved in the etiology of this malignancy. The results of these and future studies will continue to combat this rare malignancy.

## Author Contributions

WB conceived of the paper. DM contributed data. SH, SA, SG, BH, and WB contributed to the initial drafting and data synthesis of the manuscript. All authors contributed to and reviewed the final manuscript.

## Conflict of Interest

The authors declare that the research was conducted in the absence of any commercial or financial relationships that could be construed as a potential conflict of interest.
